# Attitudes of dentists and patients towards the introduction of artificial intelligence in dentistry

**DOI:** 10.25122/jml-2024-0382

**Published:** 2025-05

**Authors:** Iliyan Iliyanov Kostov, Greta Roussanova Yordanova

**Affiliations:** 1 Department of Business Administration, International Business School, Botevgrad, Bulgaria; 2 Department of Orthodontics, Faculty of Dental Medicine, Medical University-Sofia, Sofia, Bulgaria

**Keywords:** artificial intelligence, digitalization of dental practice, dental diagnosis, perception, AI: Artificial Intelligence, AR: Augmented Reality, VR: Virtual Reality, CBCT: Cone Beam Computed Tomography

## Abstract

This study evaluated the attitudes of dental professionals and patients regarding the use of artificial intelligence (AI) in dental practice. A survey was conducted among dentists, dental students, and patients to assess their trust in AI-generated diagnoses—whether partially or entirely AI-based—and in the direct involvement of AI in treatment. The collected data were statistically processed and analyzed. While approximately 45% of participants across all groups supported the use of AI software for data collection and analysis, most agreed that final diagnostic decisions should remain the dentist's responsibility. Confidence in fully AI-generated diagnoses was low, with only 3.8% of dentists, 10.4% of students, and 10.8% of patients expressing trust in such outcomes. Similarly, the level of trust in dental treatment performed by a computer-controlled dental machine was unsatisfactory. A significant difference was observed in preferences for direct dental care: 66.5% of patients favored it, compared to 79.9% of students and 77.4% of practicing dentists. This discrepancy may be attributed to concerns that AI could substantially reduce the clinical involvement of dentists and students, whereas patients fear that relying solely on AI could compromise the personalized aspect of care, requiring them to entrust their treatment entirely to machines. Hybrid solutions are models that synchronize the analytical capabilities of a dental practitioner with the data inferred by AI. The study found that doctors and patients have positive attitudes towards the introduction of AI as an auxiliary tool. Dental practices should invest in software and technological equipment that enable a new level of dental diagnosis with the aid of AI.

## INTRODUCTION

The digital transformation of dental practices and resulting business model innovations have fundamentally changed patient expectations and behavior. Digital dental medicine provides additional tools for prevention and treatment, thereby improving or restoring the quality of life for patients [1]. New technologies aid clinicians and patients in collecting, diagnosing, and analyzing paraclinical research results, as well as in creating treatment plans and producing treatment appliances or structures [2,3]. From a business perspective, the digitalization of protocols in practice enhances treatment outcomes, which in turn increases patient satisfaction [4,5]. At the beginning of a digitization process, investment costs increase; however, this increase is offset by indirect revenues and late benefits that are expressed in terms of reduced time for technical or clinical procedures, reduced rate of human error or inaccuracy, higher speed and quality of communication, predictability of results, and most importantly patient comfort [1].

The digitalization of dental practice has limitations, including the high initial costs of introducing digital technologies, the need to train a team to master new digital software and techniques, and the new level of health culture among users and patients [6,7]. Practically, not all dental procedures can be completely replaced by digital technologies, and traditional approaches are still required in some instances. However, the integration of digital technologies enables the personalization of treatment plans and dental appliances, enhancing clinical outcomes. In this context, artificial intelligence (AI) serves as a valuable tool [8-10].

Owing to the advanced algorithms and deep learning capacity of AI, its applications in dentistry can be broadly categorized into two domains: virtual and physical [11,12]. The virtual aspect is often invisible to the patient and is related to the diagnosis and storage of digital data regarding treatment. Particularly in orthodontics, artificial intelligence is utilized for automatic cephalometric analyses and tracking, 3D imaging and model analysis, as well as the planning and creation (milling or printing) of treatment appliances with great precision, accuracy, and time efficiency. The physical aspect is related to the use of robotic systems that reduce manual and less precise labor in the production of appliances, prosthetic dentures, and restorations. Orthodontic practices have evolved by incorporating intelligent robots to bend and activate arch wires, enabling humans to perform other tasks with high cognitive demands [13-16].

The advancement of AI algorithms in dentistry largely depends on the willingness of dental professionals to adopt and utilize these technologies, as their usage contributes to the continuous training and refinement of AI systems. Therefore, it is essential to investigate the positive and skeptical attitudes within the dental community. In parallel, it is essential to understand the patients' attitudes towards the development of dental services in this direction. Their satisfaction with dental treatment serves as a regulator for the future development of dental practices. The underlying hypothesis is that monitoring the attitudes of both key participants in dental care—dentists and patients—can offer valuable insights into the trajectory of digitalization in dentistry. Accordingly, this study aimed to assess the attitudes of the Bulgarian dental community and patients toward the appropriate use of artificial intelligence in dental practice, with a particular focus on their understanding of ethical boundaries.

## MATERIAL AND METHODS

### Study design and participants

A questionnaire was developed for this study, and the results served as the basis for the analyses, summaries, conclusions, and practical recommendations. The survey was anonymous and voluntary, and all participants completed it. Three different groups of respondents, including dental doctors, dental students, and patients, were included. The participants voluntarily completed a survey after the purpose of the study was explained to them. The questionnaire included clear and concise questions, each with a single-choice response format.

Definitions and explanations of key concepts were provided, especially for questions addressed to the patient group, to ensure accurate understanding. For example:

- *Final diagnosis* was defined as the synthesis of clinical and paraclinical data (e.g., X-rays, models, photographs).
- *A treatment plan* refers to the structured steps of medical interventions as outlined in established clinical protocols.
- *Treatment* indicated the actual procedures performed in the patient’s mouth.

To ensure patients had sufficient experience to evaluate changes in dental practice, a screening question was included: *“How often do you visit your dentist or dental specialist?”* The responses were as follows:

- Once every 6 months: 41.0%
- As needed: 32.7%
- Once a year: 25.9%
- Once every 2 years: 0.4%

This distribution confirmed that the majority of patients had regular dental visits, ensuring the credibility and relevance of their responses.

Participants were grouped by role (dentists, dental students, and patients), with demographic distribution summarized in Table 1.

**Table 1:** Comparative analysis of age distribution and gender differences among dentists, dental students, and patients

Groups	Age groups (years)	*n* (%)	Average ± SD	Men *n* (%)	Women *n* (%)	*P*
Dentists	18–35	16 (30.2)	30.25 ± 2.65	6 (25.3)	10 (25.0)	0.138
	36–50	12 (22.6)	43.00 ± 4.63	4 (59.0)	8 (20.0)	
	>50	25 (47.2)	57.08 ± 4.32	3 (15.7)	22 (55.0)	
Dental students	18–35	192 (100)	22.93 ± 1.58	61 (31.8)	131 (68.2)	0.001
Patients	18–35	74 (29.5)	26.31 ± 5.78	21 (25.3)	53 (31.5)	0.312
	36–50	155 (61.8)	43.06 ± 3.91	49 (59.0)	106 (63.1)	0.530
	Over 50	22 (8.8)	56.41 ± 5.24	13 (15.7)	9 (5.4)	0.007

**Group 1 – Dentists:** A total of 53 dentists (13 men, 24.5%; 40 women, 75.5%) participated, with a mean age of 45.79 ± 12.35 years (range: 25–65). The group included participants with different work experiences and specialties recruited during a national dental forum. Age distribution: 25–35 years (30.2%), 36–50 years (22.6%), and over 50 years (47.2%). Participants came from both individual and group practices across different regions.

**Group 2 – Dental students**: A total of 192 students (61 men, 31.8%; 131 women, 68.2%) from the fourth and fifth years of study participated. Their mean age was 22.93 ± 1.58 years (range: 20–32). All had entered the clinical training phase of their education.

**Group 3 – Patients:** A total of 251 patients (83 men, 33.1%; 168 women, 66.9%) with a mean age of 39.29 ± 10.29 years (range: 18–66) completed the survey.

Participants who did not complete all required fields in the questionnaire were excluded from the study.

### Data analysis

Collected data were entered and processed using IBM SPSS Statistics (version 27.0.1.0), MedCalc (version 19.6.3), and Microsoft Excel (Office 2021). Statistical significance was determined at a *P* value < 0.05.

## RESULTS

**Figure 1:**
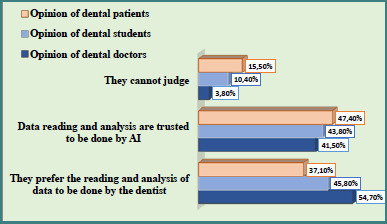
Distribution of attitudes among dentists, dental students, and patients regarding the use of AI for reading and analyzing diagnostic data in dental practice

**Figure 2:**
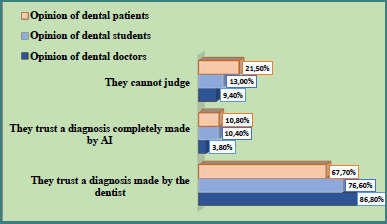
Distribution of attitudes among dentists, dental students, and patients regarding trust in diagnoses made entirely by AI versus those made by a dentist

**Figure 3:**
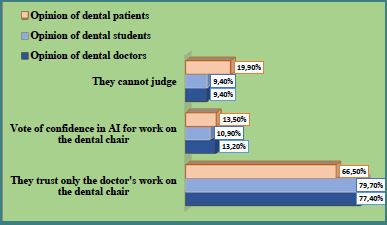
Perceptions of dentists, dental students, and patients regarding dental treatment performed by a computer-controlled (AI-driven) machine

The study examined the perspectives of three respondent groups—dentists, dental students, and patients—regarding the integration of artificial intelligence in dental diagnostics and treatment. Perceptions of trust in AI were assessed across three distinct levels:

- Level 1: Would you trust a diagnosis that relied on data obtained by AI or data whose reading or interpretation was aided by AI with specialized software (e.g., computer reading of radiographs, computer analysis of patterns, or other)? (Figure 1, Table 2)
- Level 2: Would you trust a diagnosis made entirely by AI? (Figure 2, Table 3)
- Level 3: Would you trust manipulation by a computer-controlled (AI) dental machine? (Figure 3, Table 4)

**Table 2:** Comparative analysis of responses to the question: "Do you accept that part of your dental diagnosis relies on data received, read, or interpreted by AI?"

Respondent group	Frequency	Response category		
		I would trust AI	I prefer medical work	I am not sure
Dentists (*n* = 53)	*n*	22	29	2
	%	41.5^a^	54.7^bc^	3.8^bc^
Dental students (*n* = 192)	*n*	84	88	20
	%	43.8^a^	45.8^ac^	10.4^ac^
Patients (*n* = 251)	*n*	119	93	39
	%	47.7^a^	37.1^a^	15.5^a^

The same superscript letters on the vertical lines indicate the absence of a significant difference, and the different letters indicate a significant difference (P < 0.05).

**Table 3:** Comparative analysis of responses to the question: "Would you trust a diagnosis made by AI to form your treatment?"

Respondent group	Frequency	Response category		
		I would trust AI	I prefer medical work	I am not sure
Dentists (*n* = 53)	*n*	2	46	5
	%	3.8^a^	86.8^b^	9.4^b^
Dental students (*n* = 192)	*n*	20	147	25
	%	10.4^a^	76.6^b^	13.0^b^
Patients (*n* = 251)	*n*	27	170	54
	%	10.8^a^	67.7^a^	21.5^a^

The same superscript letters on the vertical lines indicate the absence of a significant difference, and the different letters indicate a significant difference (P < 0.05).

**Table 4:** Comparative analysis of responses to the question "How would you perceive dental treatment performed by a computer-controlled (AI) dental machine?"

Respondent group	Frequency	Response category		
		I would trust AI	I prefer medical work	I am not sure
Dentists (*n* = 53)	*n*	7	41	5
	%	13.2^a^	77.4^ac^	9.4^ac^
Dental students (*n* = 192)	*n*	21	153	18
	%	10.9^a^	79.7^bc^	9.4^bc^
Patients (*n* = 251)	*n*	34	167	50
	%	13.5^a^	66.5^a^	19.9^a^

The same superscript letters on the vertical lines indicate the absence of a significant difference, and the different letters indicate a significant difference (P < 0.05).

A difference was observed among the data obtained in the three groups. Overall, approximately 45% of participants agreed that AI could be useful for data collection and analysis; however, the majority emphasized that the final diagnosis should be made by a dental professional (Table 2, Figure 1). Approval for AI-assisted diagnosis was similar across groups: 41.5% among dentists (Group 1), 43.8% among dental students (Group 2), and 47.4% among patients (Group 3). The strongest preference for maintaining the clinician’s role in diagnosis was observed among dentists (54.7%), followed by students (45.8%) and patients (37.1%). Positive attitudes toward the use of AI for data collection, analysis, and assistance in diagnosis were comparable across the three groups: 41.5% of dentists (Group 1), 43.8% of dental students (Group 2), and 47.4% of patients (Group 3) (Table 2, Figure 1). The inability to accurately judge the discussed issue was significantly greater among patients (15.5%) than dental specialists (3.8%). Among students, the rate of indecision was also relatively elevated at 10.4%.

Confidence in AI-provided dental treatment diagnosis expressed by the three groups of respondents is presented in Table 3 and visualized in Figure 2.

Confidence in diagnoses made entirely by artificial intelligence (AI) remained low across all three respondent groups (Table 3, Figure 2). Only 3.8% of dentists, 10.4% of dental students, and 10.8% of patients expressed trust in fully automated diagnostic decisions. The majority of respondents preferred diagnoses made by a human clinician, with 86.8% of dentists, 76.6% of students, and 67.7% of patients selecting this option. The relative proportions of participants who gave these responses were not statistically different. Uncertainty regarding this question was significantly more prevalent among patients (21.5%) than among dental professionals (9.4%) and students (13.0%), though the difference between the latter two groups was not statistically significant.

The level of trust in dental treatment performed by a computer-controlled dental machine (AI) among the three groups of respondents is presented in Table 4 and Figure 3. The three types of respondents demonstrated statistically equal confidence in a dental treatment performed by a computer-controlled dental machine (AI), although the confidence was low, as shown in Table 4. While differences in acceptance levels were not statistically significant, trends were notable: 66.5% of patients preferred treatment performed by a human clinician, compared to 77.4% of dentists and 79.9% of students (Figure 3). The inability to make a judgment on the issue under consideration was statistically significantly greater for patients (19.9%) than for students (9.4%) but not for dental professionals (9.4%). However, the relative proportions of participants in each group expressing this opinion did not statistically differ (Table 4).

## DISCUSSION

The digitalization process of dental practice encompasses all stages of treatment, including diagnosis, planning, production of treatment appliances and dentures, follow-up of the results, and, in the case of orthodontic treatments, retention of the results. In their study, Mörch *et al*. reported that a total of 53 different applications of AI in dentistry were identified, involving most dental specialties [17]. Hybrid solutions are being increasingly utilized in daily practice to enhance diagnostic accuracy, inform treatment planning, and optimize patient management. These are models that synchronize the analytical capabilities of a dental practitioner with data inferred by AI. Artificial intelligence leverages the power of deep learning to extract dental data rapidly and applies algorithms to analyze radiological images or digital models. This ability is of great importance in dentistry, where the complex relationships between various tooth structures and their overall restoration are dependent on the accuracy of the diagnosis.

Approximately 45% of respondents across all three groups supported the use of specialized AI software for data collection and analysis in the diagnostic process. However, they emphasized that a qualified dentist should make the final diagnosis. Trust in diagnoses made entirely by AI was statistically low across all groups: 3.8% among dentists, 10.4% among dental students, and 10.8% among patients. These findings align with a study by Stai *et al*., which reported comparable levels of trust in AI-generated and clinician-made diagnoses. Notably, participants in that study expressed higher trust in AI for cancer diagnosis; however, more than half (55%) reported discomfort with automated robotic surgery [18].

The level of trust in dental treatment performed by a computer-controlled dental machine (AI) was unsatisfactory. Statistically, the percentage of patients who preferred direct dental work (66.5%) was significantly lower than that of students (79.9%) but closer to that of a practicing dentist (77.4%).

Naturally, differences emerged in the responses and perceptions across the three surveyed groups. For dental professionals (Group 1) and dental students (Group 2), the use of AI was seen as a factor that could significantly reduce the scope of clinical work. In contrast, patients (Group 3) perceived AI as the primary agent in disease diagnosis and treatment execution, raising concerns about entrusting their bodies to machine-led interventions.

To further assess these perceptions, one study used the Artificial Intelligence Perceptions Scale (AIPS), which was developed to evaluate professional attitudes toward AI and their relationship with personal, occupational, and public perceptions [19]. The scale is psychometrically robust, demonstrating reliability and validity as the emotional or unconscious perception of AI can impact its adoption and integration in various fields.

One limitation of the current study is the relatively small number of surveyed dentists compared to the patient cohort. However, this is partially mitigated by the inclusion of a substantial number of dental students (Group 2), who represent the future of the profession. These students are being trained within a digital environment and are expected to work in settings where the controlled use of AI in dental diagnostics and robotic treatment becomes increasingly common.

The analysis revealed that a significant proportion of dental practitioners prefer to rely on their clinical judgment when making diagnoses (Figures 1 and 2), reflecting their sense of responsibility for diagnostic accuracy and treatment outcomes. Nonetheless, when it comes to AI-assisted interpretation, such as software reading of radiographic images, approximately 50% of dentists expressed trust in using AI as a supportive tool (Figure 1). This collaboration between human expertise and artificial intelligence is no longer theoretical; it is becoming a practical reality, especially in applications such as cone-beam computed tomography (CBCT) interpretation and cephalometric analysis [20,21]. This aligns with current trends, which indicate that both society and dental professionals are increasingly embracing digital systems and software as clinical decision aids. Conversely, the integration of artificial intelligence models into clinical practice requires an additional assessment of profitability, reliability, and preparation of teams for this environment. Therefore, the dental community remains skeptical about these innovations.

The survey revealed that all three groups of respondents had similar levels of confidence in a diagnosis made entirely by AI, at approximately 10%. Therefore, AI can currently be used as an additional reassuring tool that supports and reinforces the authority of dentists in diagnosing and prescribing treatment protocols. A diagnosis made by AI that confirms the dentist's opinion will be a sufficient motive for confidence in the accuracy of the diagnosis and will reduce the percentage of patients who seek a second opinion before undergoing treatment.

From an ethical standpoint, the use of AI in diagnostics raises additional concerns. Much of the data used by AI systems is sourced from the internet, where the presence of inaccurate or misleading information, referred to as 'garbage in', poses a risk to the reliability of AI-generated outcomes. This type of 'fake information' alters the environment and distorts the actual results. Despite calls from numerous organizations to ensure the integrity of online medical data, complete control over these sources remains elusive. A study by Mörch *et al*. identified 45 distinct ethical issues related to the use of AI in dentistry, reported across 22 studies (12.4%), underscoring the complexity of this challenge [17]. Dentists are not ready yet to be replaced by AI-controlled robotic machines or to be replaced by AI in their direct medical work (Figure 3). The attitudes of both students and patients toward the integration of AI in dental practice were notably aligned. However, the full realization that AI and digital technologies will revolutionize dental medicine appears to require more time. The results suggest that Bulgarian patients and dentists are not yet fully prepared to adopt advanced innovations, such as augmented reality (AR) and virtual reality (VR), in clinical settings. Augmented reality is defined as an interactive technological system that overlays animated or digital information onto the real-world environment. In dentistry, AR can be applied in scenarios such as robotic caries excavation, where the dentist remotely operates a dental machine via a computer interface while the patient observes the process in real-time, actively participating in the coordination of treatment [22]. The patient can observe the process in real time and be part of the team that coordinates the work. A more real-world application of AR/VR can be observed in simulations that utilize a 3D digital model, which superimposes STL files of the intraoral scan, segmented CBCT, and face scan. This modeling facilitates the visualization of treatment options (VTO), thereby enhancing interaction and communication with patients. Additive manufacturing of orthodontic appliances, prosthetic dentures, or restorations with complex geometric designs is currently used in dentistry [23]. 3D printing or milling enables dentists to produce a device immediately after completing the virtual design, facilitating planning and communication between the dentist and the patient. The capabilities of AI suggest that new treatments can be generated based on the analysis of existing treatments [24,25]. Simultaneously, we must note that technological progress highlights challenges for the dental society. Digital technologies and the assistance of AI help to individualize dental and orthodontic treatments.

The study found positive attitudes among both groups of participants in the dental treatment process (doctors and patients) towards the introduction of AI as an auxiliary tool. The participation of AI in the diagnostic process is the more realistic prospect for both groups of respondents. Therefore, dental practices should invest in software and technological equipment that enable a new level of dental diagnosis with the aid of AI. The process of robotizing manual dental services is still in the future, but it is not far off.

## CONCLUSION

The use of AI requires adopting a new routine, which demands time, effort, and a readiness to accept new technologies. This will increase the level of clinical dental work and the number of new patients while simultaneously reducing human activity and the possibility of human error. Dentists and patients, as key partners in the treatment process, often hold differing perceptions of AI due to their unequal positions, differing motivations, and varying levels of social and professional preparedness. The future of dental practice must, therefore, accommodate the expectations of a new generation of patients by offering services that are both technologically advanced and personally acceptable, whether delivered in a traditional or virtual setting. A dentist and their team must maintain developed skills to manage the communication process with patients, stay up-to-date with technological and innovative developments, and think strategically, all of which burden dentists with numerous responsibilities that require specific skills and knowledge. Artificial intelligence and innovative technologies can aid dentists in developing complex and personalized dental treatment plans. The hybrid combination of AI and dentists will increase the success rate and treatment and diagnostic efficiency.

## Data Availability

The data that support the findings of this study are available from the corresponding author upon reasonable request.
